# Micro-Mar: a database for dynamic representation of marine microbial biodiversity

**DOI:** 10.1186/1471-2105-6-222

**Published:** 2005-09-09

**Authors:** Ravindra Pushker, Giuseppe D'Auria, Jose Carlos Alba-Casado, Francisco Rodríguez-Valera

**Affiliations:** 1 Evolutionary Genomics Group, Universidad Miguel Hernández, Apartado 18, 03550 San Juan de Alicante, Alicante, Spain

## Abstract

**Background:**

The cataloging of marine prokaryotic DNA sequences is a fundamental aspect for bioprospecting and also for the development of evolutionary and speciation models. However, large amount of DNA sequences used to quantify prokaryotic biodiversity requires proper tools for storing, managing and analyzing these data for research purposes.

**Description:**

The Micro-Mar database has been created to collect DNA diversity information from marine prokaryotes for biogeographical and ecological analyses. The database currently includes 11874 sequences corresponding to high resolution taxonomic genes (16S rRNA, ITS and 23S rRNA) and many other genes including CDS of marine prokaryotes together with available biogeographical and ecological information.

**Conclusion:**

The database aims to integrate molecular data and taxonomic affiliation with biogeographical and ecological features that will allow to have a dynamic representation of the marine microbial diversity embedded in a user friendly web interface. It is available online at .

## Background

The global oceanic ecosystem is highly dependent on the activity of its large population of prokaryotes. However, their small size, relatively diluted environment and reluctance to be grown in pure culture make marine prokaryotes also one of the less known group of microbes. During the last decade, PCR based approaches have produced an enormous amount of information in this field, mostly based on the 16S rRNA genes due to their accepted taxonomic relevance [1]. Other genes such as *rpoB *[2] and *recA *[3] have started to be used on the grounds of their evolutionary stability. In recent years the sequencing of large insert libraries such as BACs or Fosmids have produced an important additional source of information. Last year, the Whole Genome Shotgun (WGS) applied to the Sargasso Sea produced a sequence database of 1.045 billion base pairs [4]. Still, many databases deal with only 16S or ITS sequences. For example, the Ribosomal Database Project counts 101632 entries corresponding to 16S sequences [5] and RISSC has more than 1600 entries corresponding to 16S-23S ribosomal spacer sequences [6]. While genomic sequences submission is continuously growing, there is not a clear correspondence in the improvement of analytical power of this enormous amount of information.

Micro-Mar is a novel database storing publicly available marine prokaryotes sequences along with their biogeographical (sampling site, latitude, longitude etc.) and ecological information (depth, temperature, salinity etc.). Each entry represents an individual marine prokaryote with one or more DNA sequences coming from a particular sampling location and depth. The database aims not only to provide a collection of marine prokaryotes data, but also a research tool to relate microbial biodiversity with its environment, opening possibilities for studying adaptations at the level of the microbial community, designing water management strategies, pollution detection or marine productivity prediction.

## Construction and content

In order to retrieve marine prokaryotes sequences from the NCBI [7], *ad hoc *queries were used. Moreover manual sequence searches were also carried out. All the sequences obtained were downloaded in GenBank format. Some of the details, such as geographic origin, depth, temperature etc., were obtained manually (if not available) by searching within the publications or by direct interaction with the authors. Type of entry indicates whether a sequence comes from offshore, inshore or sediments and a PCR product, a cloned DNA product or an isolated strain. A BLAST [8] search against Micro-Mar was performed in order to get the closest marine prokaryotes sequence and also the closest taxonomic unit (generally a pure culture). Top fifty BLAST hits are also available on the webpage to give more idea about the complete similarity profile for a particular sequence. Top fifty BLAST hits to whole NCBI nucleotide sequence database are also reported. The complete dataset was loaded in to MySQL [9] relational tables. Micro-Mar uses LAMP: The Open Source Web Platform [10]. Geographic Information System (GIS) uses JpGraph library [11] to display different sampling locations on a world map. All the web pages follow HTML 4.01 standard and use CSS for consistent styling.

## Utility

There are five major options available in the Micro-Mar database: (i) search, (ii) GIS, (iii) local BLAST, (iv) MMSeqUp and (v) forum.

### Search

The search option provides an interface for a large number of queries to the database. It can be used to search the database for 5S rRNA, 16S rRNA, ITS, 23S rRNA or CDS sequences along with various biogeographical and ecological parameters. The results can be either in tabular format or in a world map showing different sampling sites through GIS. Each entry is linked back to other databases such as NCBI for more information. A number of entries can be selected to analyze further along with the given sequences by aligning using CLUSTALW [12] and a tree can be created using PHYLIP [13] to see the phylogenetic position of submitted sequences against the Micro-Mar sequences. Alignment files (PHYLIP format) and tree files (Newick tree format, Postscript and PDF) are also available for download.

### GIS

The GIS option provides an interface for selecting a particular sampling location on the world map and getting all the sequences from that location and their details. Furthermore, for a selected region on the map, the following information can be obtained, i) taxonomy report: taxonomic details at different levels (domain, phylum, class, order, family and genus); ii) depth report: a plot showing number of sequences vs depth; iii) biodiversity report: a list of organisms found; iv) get all entries and v) advanced search. The reports allow to retrieve sequences corresponding to a particular taxonomy, depth or biodiversity.

### Local BLAST

The local BLAST option can be used to do a BLAST search against Micro-Mar database to get the most similar sequences to the submitted sequences. The results can be either in default BLAST format or in a tabular format. All the hits can be selected and displayed on the world map using GIS and all the GIS features discussed above can be used. Selected sequences can be downloaded in FASTA Format and also analyzed further by aligning and creating a tree along with the given sequences. Alignment and tree files can also be downloaded in different formats as described in the search option.

### MMSeqUp

MMSeqUp facilitates online sequence submission to the Micro-Mar database. It allows users to upload a file containing new marine prokaryotes sequences. Related biogeographical and ecological parameters can also be submitted on the webpage.

### Forum

An online forum powered by PHPBB [14] has been created for the following tasks: (i) FAQs: a compilation of frequently asked questions, (ii) suggestions, (iii) discussion: open discussion and iv) feedback: comments on the Micro-Mar web interface v) What's new: Recent developments in the database.

## Discussion

**Figure 1:**
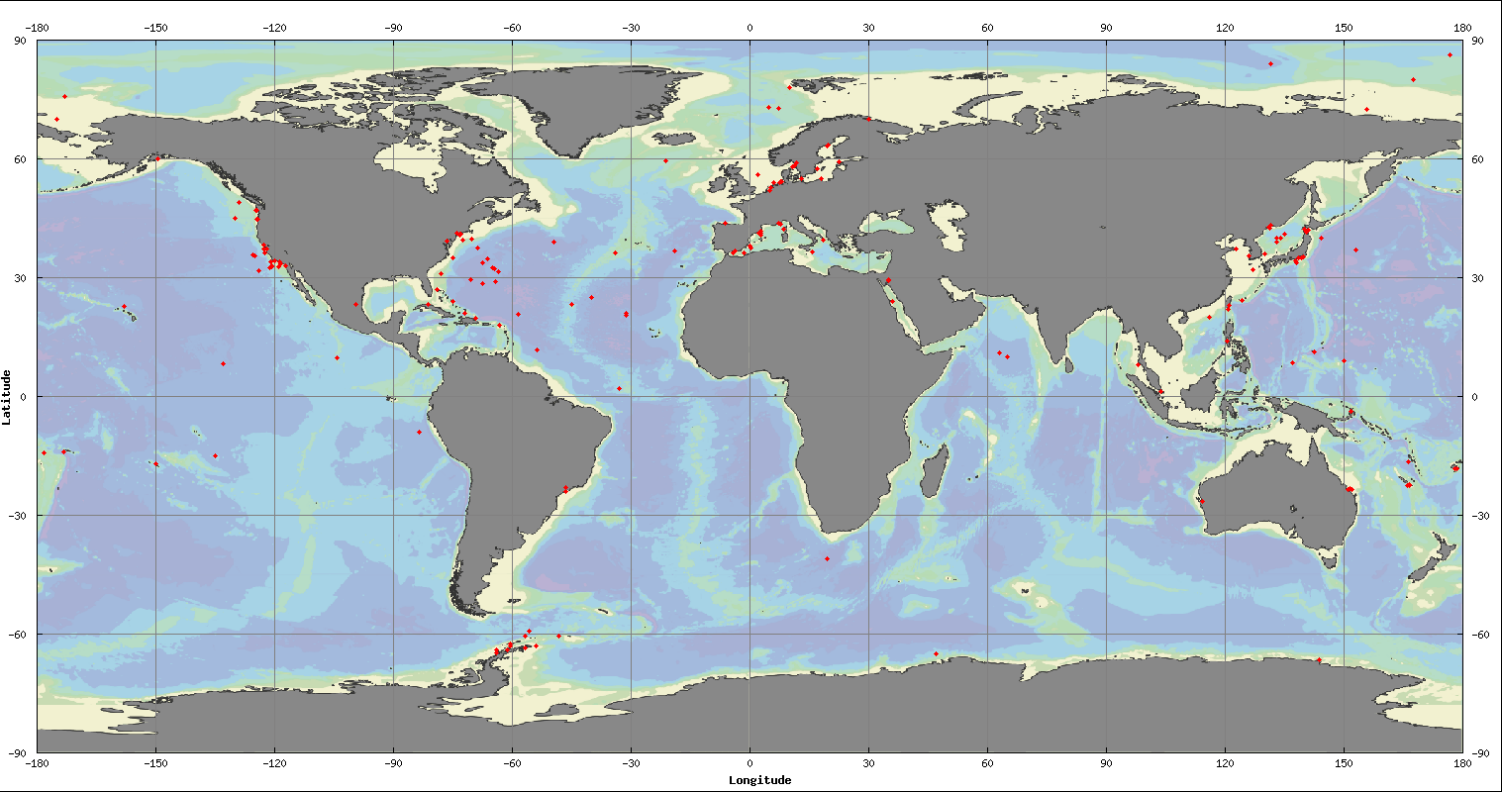
**World map showing all sampling locations**. A figure showing 192 different geographic locations widespread on the world oceanic map from Arctic to Antarctic environment.

Micro-Mar currently has 8187 entries consisting of 11874 sequences including 5693 16S rDNA, 2177 ITS, 170 23S rDNA, 3448 CDS and one 5S rDNA. Micro-Mar sequences cover 192 different sampling sites widespread on the world oceanic map from Arctic to Antarctic environments representing almost all the oceans (Figure 1). Inshore and offshore representatives are also present demonstrating a wide range of depth, going from surface water (0.5 m) to the deepest of the Mariana Trench (10898 m). The entries fall in two superkingdoms of Archea (959) and Bacteria (6351). There is also a group of 877 unclassified entries. Out of 8187 entries, 7672 entries i.e. more than 93% of entries have complete geographic information available in the database. Similarly more than 85% of entries have depth information available. All the entries have taxonomic details linked back to the NCBI taxonomy database [15]. The details of taxonomic distribution at phylum and class level are summarized in Table 1 [see Additional file 1]. As expected in the marine environment, the biggest taxonomic group is represented by the Proteobacteria with all the 5 classes (*α*, *β*, *γ*, *δ *and *ε*) as shown in the Table 2 [see Additional file 1].

## Conclusion

The creation of Micro-Mar database is an initiative towards cataloging all the information related to marine prokaryotes collected during the lasts two decades and providing an interface that will help the scientific community to do comparative analyses of marine prokaryotes sequences and make it amenable for biogeographical and ecological analyses. The database is updated every week to include the most recent marine prokaryotic sequences. In near future, more samples from extreme environments will be integrated in the database to improve the analytical power and the biodiversity range. As more and more entries are incorporated, it will be possible to correlate accurately the bacterial biodiversity with biogeographical and ecological parameters giving a global overview of the various aspects of the biodiversity within the oceans. In order to achieve this, it is encouraged that scientists include more information about biogeographical and ecological parameters while submitting their sequences to various public databases.

## Availability and requirements

The database is available at . A latest web browser with JavaScript enabled is required to use it.

## List of abbreviations

GIS – Geographic Information System

LAMP – Linux + Apache + MySQL + PERL/PHP/Python

## Authors' contributions

FRV conceived the study and the general design of Micro-Mar. RP and GD drafted the manuscript. GD and JCA did the specific design, structure development and data input. All the informatics applications were designed and developed by RP. All authors read and approved the final manuscript.

## Supplementary Material

Additional file 1 **Taxonomic distribution of Micro-Mar sequences** Table-1 shows taxonomic distribution of sequences from different domains at "Class" level and Table-2 shows taxonomic distribution of sequences from Proteobacteria class at "Family" level. Click here for file
