# Supplementary material for: Micro-Mar: a database for dynamic representation of marine microbial biodiversity
Source: BMC Bioinformatics. 2005 Sep 9;6:222. doi: 10.1186/1471-2105-6-222 (PMC1242345; doi:10.1186/1471-2105-6-222)
Supplement: Additional file 1 — Taxonomic distribution of Micro-Mar sequences Table-1 shows taxonomic distribution of sequences from different domains at "Class" level and Table-2 shows taxonomic distribution of sequences from Proteobacteria class at "Family" level. [file 1471-2105-6-222-S1.PDF]

Table 1. Taxonomic distribution of Micro-Mar sequences from Bacteria and Archaea domain at "Class" level.

Domain	Phylum	Class	16S	ITS	23S	CDS	U[a]	Total
Archaea	Unclassified	Unclassified	536					536
	Crenarchaeota	Unclassified	135	224	49	42		450
		Thermoprotei	11					11
	Euryarchaeota	Unclassified	68					68
		Halobacteria	32					32
		Methanomicrobia	2					2
**Total Archaea**			**784**	**224**	**49**	**42**		**1099**
Bacteria	Unclassified	Unclassified	841	517	1			1359
	Actinobacteria	Actinobacteria	110	4	2			116
	Aquificae	Aquificae	1					1
	Bacteroidetes	Unclassified	255	54				309
		Flavobacteria	34					34
		Sphingobacteria	48	1	1			50
	Chlamydiae	Chlamydiae	1					1
	Chlorobi	Unclassified	5					5
	Chloroflexi	Chloroflexi	44					44
	Cyanobacteria	Unclassified	165	7	5	1857		2035[b]
	Fibrobacteres	Fibrobacteres	6					6
	Firmicutes	Unclassified	43	1				44
		Clostridia	5					5
	Fusobacteria	Fusobacteria	1					1
	Nitrospirae	Unclassified	82					82
	Planctomycetes	Planctomycetacia	60					60
	Proteobacteria	Unclassified	23	5	5	2		35
		Alphaproteobacteria	957	515	99	1		1572
		Betaproteobacteria	43					43
		Deltaproteobacteria	257	54				311
		Epsilonproteobacteria	36					36
		Gammaproteobacteria	1425	794	7	13	1	2240
	Spirochaetes	Spirochaetes	9					9
	Verrucomicrobia	Unclassified	17	1	1			19
		Verrucomicrobiae	2				1	3
**Total Bacteria**			**4470**	**1953**	**121**	**1873**	**2**	**8420**

a - Unknown sequence type

b - Total is more than the sum of 16S rRNA, 23S rRNA, ITS, CDS and unknown sequences because of presence of one 5S rRNA sequence in the database.

Table 2. Taxonomic distribution of Micro-Mar sequences from Proteobacteria class at "Family" taxonomy

a - Unknown sequence type

Class	Order	Family	16S	ITS	23S	CDS	U[a]	Total
Alphaproteobacteria	Unclassified	Unclassified	524	221	87	1		833
	Caulobacterales	Caulobacteraceae	6					6
	Parvularculales	Parvularculaceae	2					2
	Rhizobiales	Unclassified	1					1
		Aurantimonadaceae	2					2
		Hyphomicrobiaceae	1					1
		Phyllobacteriaceae	1					1
		Rhizobiaceae	2					2
	Rhodobacterales	Rhodobacteraceae	106	2	2			110
	Rhodospirillales	Rhodospirillaceae	2					2
	Rickettsiales	Unclassified	283	292	10			585
	Sphingomonadales	Sphingomonadaceae	27					27
**Total Alphaproteobacteria**			**957**	**515**	**99**	**1**		**1572**
Betaproteobacteria	Unclassified	Unclassified	27					27
	Burkholderiales	Alcaligenaceae	1					1
		Comamonadaceae	1					1
	Nitrosomonadales	Nitrosomonadaceae	12					12
	Rhodocyclales	Rhodocyclaceae	2					2
**Total Betaproteobacteria**			**43**					**43**
Deltaproteobacteria	Unclassified	Unclassified	255	54				309
	Myxococcales	Unclassified	1					1
		Nannocystaceae	1					1
**Total Deltaproteobacteria**			**257**	**54**				**311**
Epsilonproteobacteria	Unclassified	Unclassified	35					35
	Campylobacterales	Campylobacteraceae	1					1
**Total Epsilonproteobacteria**			**36**					**36**
Gammaproteobacteria	Unclassified	Unclassified	762	298	6	5	1	1072
	Aeromonadales	Aeromonadaceae	1					1
	Alteromonadales	Unclassified	8	5				13
		Alteromonadaceae	187	139	1			327
		Colwelliaceae	48	16				64
		Idiomarinaceae	3					3
		Moritellaceae	3					3
		Pseudoalteromonadaceae	178	171				349
		Psychromonadaceae	7					7
		Shewanellaceae	49	33				82
	Chromatiales	Unclassified	54	63				117
		Chromatiaceae	1					1
	Enterobacteriales	Enterobacteriaceae	3			1		4
	Oceanospirillales	Unclassified	10					10
		Alcanivoraceae	2					2
		Halomonadaceae	10					10
		Oleiphilaceae	1					1
	Pseudomonadales	Moraxellaceae	12	1				13
		Pseudomonadaceae	11					11
	Thiotrichales	Unclassified	31	68				99
	Vibrionales	Vibrionaceae	42			7		49
	Xanthomonadales	Xanthomonadaceae	2					2
**Total Gammaproteobacteria**			**1425**	**794**	**7**	**13**	**1**	**2240**
